# Uptake and acceptability of assisted and unassisted HIV self-testing among men who purchase sex in brothels in Indonesia: a pilot intervention study

**DOI:** 10.1186/s12889-020-08812-4

**Published:** 2020-05-19

**Authors:** Luh Putu Lila Wulandari, John Kaldor, Rebecca Guy

**Affiliations:** 1 The Kirby Institute, University of New South Wales, Level 6 Wallace Wurth Building, UNSW Kensington Campus, Sydney, NSW 2052 Australia; 2 Department of Public Health and Preventive Medicine, Faculty of Medicine, Udayana University, Denpasar, Indonesia

**Keywords:** HIV testing, HIV self-testing, Men who purchase sex, Clients of sex workers, Scale-up, Lay workers, Indonesia

## Abstract

**Background:**

Along with sexual partners of other high-risk groups, men who purchase sex (MWPS) represented 18% of new HIV diagnoses worldwide in 2018. They are therefore an important population for HIV prevention globally. Despite very low HIV testing coverage among MWPS in many countries, the role of HIV self-testing to increase testing coverage has not been explored. We, therefore, conducted a pilot intervention study to evaluate the uptake and acceptability of assisted and unassisted HIV self-testing among MWPS in Indonesia.

**Methods:**

MWPS attending seven brothels in Bali between December 2017 and January 2018 were recruited by lay health providers to participate in a brief health survey, and then invited to have a HIV self-test (assisted or unassisted) with an OraQuick® ADVANCE Rapid HIV-1/2 Antibody Test and complete a post-test acceptability survey.

**Results:**

A total of 292 men completed the health survey (response rate: 70%) and 188 (64.6%) accepted HIV self-testing. Of these men, 13.3% had ever tested for HIV and 58.9% reported condom use at their last sexual encounter with a brothel-based female sex worker. Nearly all men (98.9%) who accepted a HIV self-test preferred assisted HIV self-testing – of whom 83.9% preferred to be fully assisted and 16.1% opted to be partially assisted and read their results privately. Of the men who accepted the test and showed the result to the lay health providers, 4 (2.1%) received reactive results. Linkage following HIV self-test is a concern, as none of the four men with a reactive result attended HIV testing at the recommended referral HIV testing clinic over a two-month follow-up period.

**Conclusions:**

This study is the first to investigate the acceptance of HIV self-testing when offered to MWPS in brothels by lay health providers. The high uptake of HIV self-testing suggests that this testing model is acceptable and could increase the very low HIV testing coverage among MWPS. The strong preference for fully assisted HIV self-testing highlights the importance of involving lay health providers in future testing programs. When scaling up HIV self-testing programmatically, strategies to improve linkage-to-care should be considered and evaluated.

## Background

In 2006, it was estimated that between 9 and 10% of the male population in many regions globally had purchased sex in the last 12 months [1]. Men who purchase sex (MWPS) are considered to be a key population for HIV prevention because of their increased risk of HIV acquisition through sexual contact with female sex workers (FSWs), who have elevated infection rates in a number of countries [2, 3], with global data in 2018 revealed that FSWs have 21 times higher risk of acquiring HIV than adults aged 15–49 in population [4]. Around 18% new reported HIV cases in almost all regions globally in 2018 were among MWPS and sexual partners of high-risk groups [5]. Several studies have shown higher HIV prevalence among MWPS than among corresponding male populations [6–15]. Furthermore, in many Asian countries, MWPS represent the largest single population at elevated risk of HIV [16]. Also, many MWPS have non-sex worker partners (wives, girlfriends) [17, 18], so there is potential for onward HIV transmission.

There is increasing focus globally on HIV treatment as prevention among key populations, based on studies showing both strong individual-level and public health benefits [19–21]. HIV treatment reduces the risk of onward HIV transmission to effectively zero [21, 22] and the risk of morbidity and mortality for people [20]. UNAIDS has set a target of 90% of people living with HIV being aware of their infection status [23, 24]. In response, in 2015, the World Health Organization (WHO) released guidelines recommending lay providers or community health workers should provide HIV testing services to those unable or reluctant to seek facility-based HIV testing [25] and HIV self-testing as an additional approach to HIV testing [26], reflecting accumulating evidence on the impact, acceptability and cost-effectiveness of this strategy [27–34]. HIV self-testing offers promise due to its practicality, convenience, non-invasive, private and confidential nature [28, 35, 36]; and has the potential to overcome barriers associated with conventional, clinic-based HIV testing such as distance and stigma [37–39]. HIV self-tests could be particularly beneficial for populations, such as MWPS, who will generally conceal their sexual activity as it places them at risk of stigma or even legal sanctions [40, 41].

In Indonesia, with a HIV prevalence among the male adult population aged 15–49 years of 0.5% in 2018 [42], a modelling study estimated that in 2016 there were 5.2 million MWPS [43], who had transactional sex with around 227 thousand FSWs [43], 8% of whom were living with HIV [44]. Yet, surveys have found less than 10% of MWPS in Indonesia have ever been tested for HIV [45, 46]. In Indonesia, HIV testing can be accessed through Voluntary Counselling and Testing (VCT) services at community health services, private clinics, or public and private hospitals. As of 2019, there were at least 6924 VCT services which reported testing activity to the Ministry of Health [47]. Recent qualitative research conducted among MWPS in Indonesia, however, identified a range of barriers to accessing traditional HIV testing modalities including embarrassment in asking for a test or being seen at the VCT clinic, fear of the invasive test, and inconvenience in terms of time and distance to the clinic [37, 48]. The interviews also revealed a strong interest in HIV self-tests to overcome these barriers [37, 48].

Despite the call to introduce HIV self-tests to improve HIV testing coverage in key populations [25, 26], there have been no demonstration projects among MWPS particularly when HIV self-testing is offered by lay health providers at brothels. There have been numerous studies of HIV self-testing conducted among other risk and marginalised populations such as FSWs [49–51] and men who have sex with men [52, 53], but findings from these key populations cannot be applied to MWPS. Further, only a few studies have compared unassisted with assisted or supervised HIV self-testing [54–58] to allow for the identification of strategy which is preferable. In this study, we evaluated the acceptance of assisted and unassisted HIV self-testing when offered to MWPS in brothels in Indonesia by lay health providers.

## Methods

### Study design

We conducted a pilot intervention study of HIV self-testing among MWPS at brothels in Bali from 1 December 2017 to 31 January 2018. The CONSORT 2010 checklist of information to include when reporting a pilot or feasibility trial was used to guide the development of this report [59].

### Setting

Bali has the sixth-highest HIV cases reported to the Ministry of Health from January to March 2019 in Indonesia [60]. All seven brothels in Denpasar Bali, often called complexes and recognized by health officials as operating in the area, and have been involved in previous HIV interventions targeting FSWs, were selected for the study. Brothels were located in complexes, with each brothel managed by a manager or pimp (a person who arranges the sex transaction with the potential clients and take a portion of the clients’ payments). The number of FSWs in each complex varies according to the size of the brothel but ranges from 12 to 155 [61].

### Recruitment

Participants were recruited by ten lay providers/workers from Yayasan Kerti Praja (YKP), a non-government organization (NGO) providing HIV clinical and prevention services to FSWs [62]. The lay workers’ age ranged from 20 to 40 years old. Most of them have been working with the NGO for more than five years, have been conducting various HIV outreach activities at brothel settings, and have been trained on various HIV capacity building activities, including intensive VCT counsellor training. The ten lay providers (six men and four women) involved in the study worked alone in each brothel selected at a given time.

MWPS can also include men who pay for sex with men/transgender people, but this paper only considers those who pay for sex with women or FSWs because the recruitment location was brothels where FSWs see their male clients. The men were first invited to participate in a brief health survey and then invited to have a HIV self-test and complete a post-test acceptability survey. The lay workers received training in the study procedures and conducting the HIV self-test. During the study period, they approached all men who were waiting in the brothel areas and asked if they would be willing to participate in a health survey. The lay workers were stationed in the brothels every day during the study period for about eight hours, from 11 am to 1 am. There were no posters or resources about HIV self-test displayed at the brothels.

**Fig. 1:**
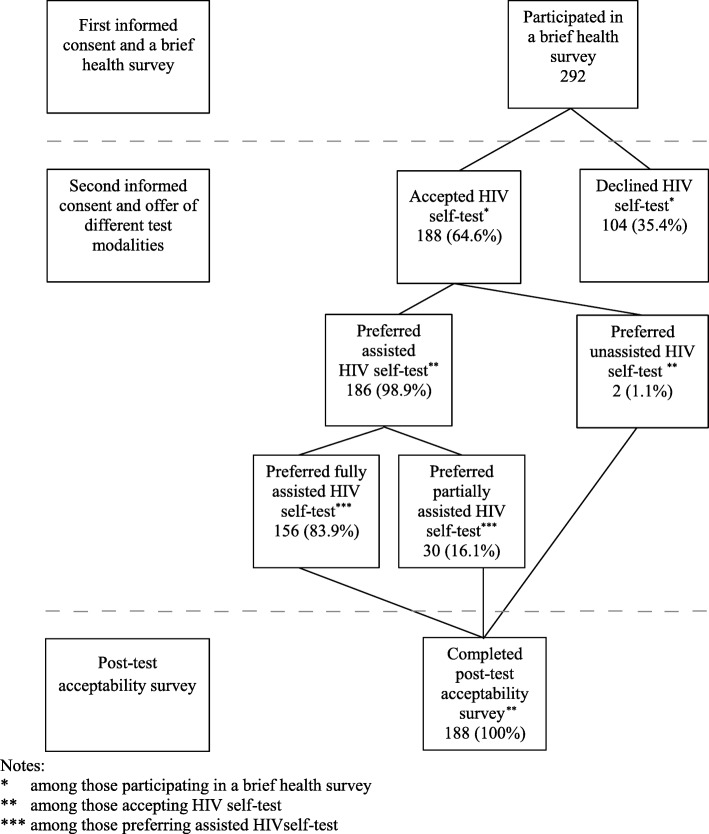
Flow chart of the study procedure and acceptance of different HIV self-test modalities

The flowchart of the study procedure can be seen in Fig. 1. Those who were willing to participate in the health survey were provided with the first informed consent prior to the survey. Once the men completed the health survey, men were offered a HIV self-test. If they agreed to participate in the HIV self-test procedure, they would be provided with the second informed consent. Finally, they would complete the post-test survey once they completed the HIV self-test procedure.

### Eligibility and consent

Men were eligible to participate in the health survey if they were: older than 18, had ever had sexual contact with FSWs, were able to communicate in Bahasa Indonesia, and were willing to provide written informed consent.

### Health survey

To improve the response rate, the health survey was introduced to clients not as specifically focused on HIV. However, the lay workers explained that the survey included questions around socio-demographic, sexual behaviours and HIV testing history. The questions were asked by the lay workers, and the interview took about 5–10 min. The decision to use an interviewer-administered, rather than a self-completed questionnaire, was based on lower education levels of the participants ascertained in a previous survey [45].

### HIV self-test

Following the health survey, men were offered a HIV self-test. For those who agreed, the lay workers explained and demonstrated how to perform the test and interpret the results, and men were offered assisted or unassisted HIV self-testing. Assisted HIV self-testing had two options: 1) Fully assisted: lay workers supervised the men while they conducted the HIV self-test onsite and interpreted the results; or 2) Partially assisted: men would conduct the HIV self-test onsite with lay workers’ supervision and interpret the result independently, but could choose to show the results to the lay worker. Unassisted HIV self-testing involved men taking the kit and performing the test offsite; and if they were willing to show the results to the lay workers, the men were asked to return the used test kit in a sealed envelope to the YKP, or take the picture of the used test kit and send it to the lay worker through text message. These multiple options were offered as qualitative research in this setting had revealed that concerns about confidentiality and embarrassment were key barriers to HIV testing [37, 48]. For partially assisted testers, there was a place at each brothel where the clients can perform the test in private. Depending on clients’ preferences, they would use their own mobile phones or watches as timekeeper, or the lay workers reminded them when it was the time to read the test result.

The OraQuick® ADVANCE Rapid HIV-1/2 Antibody Test (OraQuick; OraSure Technologies, Inc., Bethlehem, PA) HIV self-test kit was used in the study. HIV self-test procedures were in accordance with the manufacturer’s instructions, which was translated into Bahasa Indonesia. For men with reactive HIV self-test results and disclosed their results to the lay workers, the lay workers would then hold a post-test counselling session, providing the men with information on HIV and HIV testing, as what the lay workers usually perform at the VCT clinic for HIV positive clients. Regardless of the results, each participant was given information about the availability of free HIV testing at the YKP clinic. Men were advised to attend the YKP clinic to confirm the result with standard rapid testing procedure with further assessment, HIV treatment and support as needed. Men could also attend other clinics if they preferred. All the used kits were stored in a box and put at the YKP clinic.

### Post-test acceptability survey

A post-test acceptability survey was conducted after the HIV self-test to discern opinions about the process and attitudes towards HIV self-testing in the future. The questions in the post-test survey were adapted from an earlier Australian study [33], with modifications made to reflect the local context and previous in-depth interviews conducted in MWPS about barriers and facilitators to HIV testing [37]. The questionnaire included questions around what the men liked about HIV self-test, what they did not like about HIV self-test, how easy the test was, the likelihood of using HIV self-test in the future, the maximum they would be willing to pay for a HIV self-test.

Likert scale items were used to assess men’s perception on ease of performance (1 = very difficult; 2 = difficult; 3 = easy; 4 = very easy); and men’s future willingness to purchase and use a HIV self-test (1 = very unlikely; 2 = unlikely; 3 = likely; 4 = very likely). The questionnaire was focus tested with ten lay workers and one pimp to confirm clarity of the questionnaire and feasibility of the HIV self-test and post-test survey procedures, with necessary changes made.

### Testing counselling and information

Pre-test and post-test counselling were offered for all men who agreed to a HIV self-test. Vouchers contained a code to be able to link it with the survey data were distributed to the men to be taken to YKP clinic for a HIV confirmation test at the clinic. Further attempts were made by lay workers to follow up clients with potentially positive results as part of standard care**.**

### Analysis

Analyses were conducted using STATA 14 (STATA Corporation, Texas, USA). The characteristics of the participants were analysed descriptively, using median and interquartile ranges (IQR), or frequency and percentages. Likert scale items were collapsed into categories: very difficult/difficult vs easy/very easy and likely/very likely vs unlikely/very unlikely. We calculated the acceptance of a HIV self-test, defined as the number of men who accepted a HIV self-test among those offered a HIV self-test by lay workers following the health survey. We also calculated the preference of the different test modalities (fully and partially assisted, and unassisted) defined as the number of men who preferred one of these options among those who accepted a HIV self-test. These outcomes were presented as the proportion with 95% confidence interval calculated.

Bivariate analysis was conducted using logistic regression, and further multivariate analysis using backward logistic regression was performed for data that was significant at less than 0.1 to identify factors associated with accepting a HIV self-test, and separately for preferring a fully assisted HIV self-test. In the multivariate analysis, only variables with the *p*-value of 0.05 were considered statistically associated with those two outcomes.

Among all participants involved in the brief health survey, we restricted the bivariate analysis of predictors of accepting a HIV self-test to participants with available data for all variables. Similarly, among all participants accepting HIV self-test, the bivariate analysis of factors associated with preferring a fully assisted HIV self-test was restricted to participants with available data for all variables. During this analysis, missing value categories for condom use during the last sexual contact with FSWs was created due to that this variable presents a high number of missing values. Ethnicity was selected as one of the covariates as in Indonesia there are around 1340 ethnic backgrounds [63], and other study has found the association between ethnicity and acceptability of HIV testing [64].

### Ethics approval

Ethics approvals were obtained from the Research Ethics Committee of Udayana University/Sanglah Hospital, Indonesia and the Human Research Ethics Committees (HRECs) of the University of New South Wales, Australia.

As it was a pilot study only, we did not register it on a trial registry.

## Results

### Characteristics of MWPS

Around 70% (292/417) of men who were approached for the health survey accepted to participate. The median age of the 292 men who participated in the health survey was 38.5 (IQR: 29.5–48) years. Just over a third of men (47.3%) had completed at least senior high school, nearly half (45.9%) were Javanese, 38% were Balinese and two-thirds (76.4%) had ever married. Men had a median number of 4 (IQR: 2–7) FSW sexual partners in the last 12 months. Around 59.7% of the men reported having used a condom at their last sexual encounter with a brothel-based FSW. Only 12.7% of the participants had ever been tested for HIV, with 5.8% of the men mentioned that they had been HIV tested in the last 12 months. Men cited multiple barriers to HIV testing at the clinic including embarrassment when asking for an HIV test (57.4%), fear of being diagnosed with HIV (46.7%), feeling healthy so not perceiving they needed a test (45.7%), fear of having blood taken (42.6%), distance from the clinic (34.6%), embarrassment being seen at a clinic (31.1%), anxiety waiting for the test results (31.8%), lack of knowledge of where to go for testing (31.5%), and concern over confidentiality (27%) (Table 1), with 241 (82.5%) men reported 2 or more barriers to HIV testing.

**Table 1:** Socio-demographic characteristics, sexual behaviours, and HIV testing history of MWPS

Characteristics of MWPS	Participated in a health survey n (%) (***N*** = 292)^	Accepted HIV self-test n (%) (***N*** = 188)^^
Median age (IQR)	38.5 (29.5–48)	38 (30–48)
Had ever married	223 (76.4)	140 (74.5)
Highest level of education		
None/primary/junior high school	154 (52.7)	96 (51.1)
Senior high school/TAFE/ degree	138 (47.3)	92 (48.9)
Ethnicity		
Balinese	111 (38)	73 (38.3)
Javanese	134 (45.9)	89 (47.3)
Other	47 (16.1)	27 (14.4)
Barriers to HIV testing at a clinic		
Embarrassed to ask for an HIV test		
No	123 (42.6)	72 (38.7)
Yes	166 (57.4)	114 (61.3)
Fear of being diagnosed with HIV		
No	154 (53.3)	112 (60.2)
Yes	135 (46.7)	74 (39.8)
I am feeling healthy		
No	157 (54.3)	107 (57.5)
Yes	132 (45.7)	79 (42.5)
Too afraid of having blood taken		
No	166 (57.4)	108 (58.1)
Yes	123 (42.6)	78 (41.9)
Embarrassed discussing sex life		
No	175 (60.5)	109 (58.6)
Yes	114 (39.5)	77 (41.4)
It’s too far		
No	189 (65.4)	119 (64)
Yes	100 (34.6)	67 (36)
Embarrassed to be seen at the clinic		
No	199 (68.9)	130 (69.9)
Yes	90 (31.1)	56 (30.1)
Anxious when waiting for results		
No	197 (68.2)	128 (68.8)
Yes	92 (31.8)	58 (31.2)
I do not know where to go		
No	198 (68.5)	130 (69.9)
Yes	91 (31.5)	56 (30.1)
I do not have time to go to the clinic		
No	199 (68.9)	125 (67.2)
Yes	90 (31.1)	61 (32.8)
Concern over confidentiality		
No	211 (73)	131 (70.4)
Yes	78 (27)	55 (29.6)
Other		
No	283 (97.9)	181 (97.3)
Yes	6 (2.1)	5 (2.7)
Had ever been tested for HIV		
No	255 (87.3)	163 (86.7)
Yes	37 (12.7)	25 (13.3)
Had been tested for HIV in the last 12 months		
No	275 (94.2)	176 (93.6)
Yes	17 (5.8)	12 (6.4)
Number of regular sexual partners* in the last year		
0	70 (24)	49 (26.1)
1 or more	222 (76)	139 (73.9)
Median number of casual sexual partners in the last year (IQR)	0 (0–1)	0 (0–1)
Median number of FSWs^#^ sexual partners in last year (IQR)	4 (2–7)	3 (2–7)
Median number of sex encounters with FSWs^#^ in last year (IQR)	6 (2–12)	6 (2–12)
Condom used at last sex with FSW^#^		
No	100 (40.3)	65 (41.1)
Yes	148 (59.7)	93 (58.9)
Location of the brothel visited		
cluster 1	59 (20.2)	42 (22.3)
cluster 2	33 (11.3)	21 (11.2)
cluster 3	26 (8.9)	16 (8.5)
cluster 4	41 (14)	27 (14.4)
cluster 5	24 (8.2)	17 (9)
cluster 6	82 (28.1)	50 (26.6)
cluster 7	27 (9.3)	15 (8)

*non-FSW

^#^ brothel-based

^Due to nonresponses on some items, columns may not sum to 292

^^Due to nonresponses on some items, columns may not sum to 188

### Uptake and factors associated with different HIV self-test modalities

Among the 292 respondents who completed the health survey, 188 (64.6%; 95%CI: 59.6–69.9%) accepted a HIV self-test (see Fig. 1). Of these 188 men, 48.9% had completed at least senior high school, 47.3% were Javanese, 13.3% had ever been tested for HIV, with 6.4% of the men mentioned that they had been HIV tested in the last 12 months, and 58.9% reported condom use at their last sexual encounter with a FSW. (Table 1). Men were less likely to accept a HIV self-test if they stated feared being diagnosed with HIV as a barrier to HIV testing at the clinic (OR 0.4; 95% CI 0.3–0.7; *p* = 0.001). On the other hand, men were more likely to accept the HIV self-test if they mentioned feeling embarrassed to ask for an HIV test at the clinic as one of the barrier to testing (OR 1.8; 95% CI 1.1–2.9; *p* = 0.030). (Table 2).

**Table 2:** Bivariate and multivariate analysis of predictors of accepting a HIV self-test

Predictors	Total (n) (***N*** = 288)	Accepted HIV self-test n (%)	Bivariate analysis of predictors of accepting a HIV self-test		Multivariate analysis of predictors of accepting a HIV self-test	
			OR (95%CI)	P value	OR (95%CI)	P value
Highest level of education						
None/primary/junior high school	153	95 (62.1)	1			
Senior high school/TAFE/ degree	135	91 (67.4)	1.3 (0.8–2.1)	0.347		
Ethnicity						
Other	46	27 (58.7)	1			
Balinese	110	72 (65.5)	1.3 (0.7–2.7)	0.425		
Javanese	132	87 (65.9)	1.4 (0.7–2.9)	0.381		
Had ever married						
Yes	222	139 (62.6)	1			
No	66	47 (71.2)	1.5 (0.8–2.7)	0.201		
**Sexual behaviour**						
Number regular sexual partners^*^ last year	288	186	0.8 (0.5–1.2)	0.214		
Number casual sexual partners^*^ last year	288	186	1.0 (0.9–1.2)	0.736		
Number FSWs^#^ sexual partners last year	288	186	1.0 (1.0–1.0)	0.211		
Condom use at last sex with FSWs^#^						
Yes	145	92 (63.5)	1			
No	99	64 (64.7)	1.1 (0.6–1.8)	0.848		
Missing	44	30 (68.2)	1.2 (0.6–2.5)	0.566		
Had ever been tested for HIV						
Yes	34	23 (67.7)	1.2 (0.5–2.5)	0.691		
No	254	163 (64.2)	1			
**Barriers to HIV testing at a clinic:**						
I do not know where to go						
No	197	130 (66)	1			
Yes	91	56 (61.5)	0.8 (0.5–1.4)	0.463		
I do not have time to go to the clinic						
No	198	125 (63.1)	1			
Yes	90	61 (67.8)	1.2 (0.7–2.1)	0.445		
It’s too far						
No	188	119 (63.3)	1			
Yes	100	67 (67)	1.2 (0.7–2)	0.532		
Embarrassed discussing my sex life						
No	174	109 (62.6)	1			
Yes	114	77 (67.5)	1.2 (0.8–2)	0.395		
Embarrassed to ask for an HIV test						
No	122	72 (59)	1		1	
Yes	166	114 (68.7)	1.5 (0.9–2.5)	**0.091**	1.8 (1.1–2.9)	**0.030**
Anxious when waiting for results						
No	196	128 (65.3)	1			
Yes	92	58 (63)	0.9 (0.5–1.5)	0.708		
Embarrassed to be seen at clinic						
No	198	130 (65.7)	1			
Yes	90	56 (62.2)	0.9 (0.5–1.4)	0.572		
Concern over the confidentiality						
No	210	131 (62.4)	1			
Yes	78	55 (70.5)	1.4 (0.8–2.5)	0.201		
Too afraid of having blood taken						
No	166	108 (65.1)	1			
Yes	122	78 (63.9)	1 (0.6–1.6)	0.844		
Fear of being diagnosed with HIV						
No	154	112 (72.7)	1		1	
Yes	134	74 (55.2)	0.5 (0.3–0.8)	**0.002**	0.4 (0.3–0.7)	**0.001**
I am feeling healthy						
No	102	53 (52)	1			
Yes	186	79 (42.5)	0.7 (0.4–1.1)	0.123		
Location of the brothel						
cluster 1	59	42 (71.2)	1			
cluster 2	33	21 (63.6)	0.7 (0.3–1.8)	0.456		
cluster 3	25	15 (61.5)	0.6 (0.2–1.6)	0.318		
cluster 4	40	27 (67.5)	0.8 (0.4–2)	0.695		
cluster 5	23	17 (70.8)	1 (0.4–3.4)	0.805		
cluster 6	81	49 (61)	0.6 (0.3–1.3)	0.192		
cluster 7	27	15 (55.6)	0.5 (0.2–1.3)	0.158		

* non-FSW

^#^ Brothel-based

Of the 188 men who accepted a HIV self-test, 186 (98.9%; 95% CI 96.2–99.9%) preferred assisted and 2 (1.1%; 95% CI 0.1–3.8%) unassisted. Of those who preferred assisted, 156 (83.9%; 95% CI 77.8–88.8%) preferred to be fully assisted and 30 (16.1%; 95% CI 11.2–22.2%) partially assisted with unsupervised reading onsite. (Fig. 1).

There was only one factor associated with preference for a fully assisted HIV self-test, which was feeling anxious when waiting for HIV test results as a barrier to HIV testing at the clinic (OR 3.8; 95% CI 1.3–11.3; *p* = 0.018). (Table 3).

**Table 3:** Bivariate and multivariate analysis of predictors of a fully assisted HIV self-test

Predictors	Total (n) (***N*** = 186)	Preferred fully assisted HIV self-test	Bivariate analysis of predictors of preferring a fully assisted HIV self-test		Multivariate analysis of predictors of preferring a fully assisted HIV self-test	
		n (%)	OR (95%CI)	P value	OR (95%CI)	P value
Highest level of education						
None/primary/junior high school	95	81 (85.3)	1			
Senior high school/TAFE/ degree	91	73 (80.2)	0.7 (0.3–1.5)	0.364		
Ethnicity						
Other	27	22 (81.5)	1			
Balinese	72	62 (86.1)	1.4 (0.4–4.6)	0.568		
Javanese	87	70 (80.5)	1 (0.3–2.8)	0.906		
Had ever married						
Yes	139	114 (82)	1			
No	47	40 (85.1)	1.2 (0.5–3.1)	0.628		
**Sexual behaviour**						
Number regular sexual partners^*^ last year	186	154	1.5 (0.7–2.9)	0.270		
Number casual sexual partners^*^ last year	186	154	0.8 (0.6–1.2)	0.250		
Number FSWs^#^ sexual partners last year	186	154	1 (1–1)	0.319		
Condom use at last sex with FSWs^#^						
Yes	92	76 (82.6)	1		1	
No	64	58 (90.6)	2 (0.8–5.5)	0.163	1.8 (0.7–5)	0.257
Missing	30	20 (66.7)	0.4 (0.2–1.1)	**0.069**	0.5 (0.2–1.2)	0.112
Had ever been tested for HIV						
Yes	23	17 (74)	0.5 (0.2–1.5)	0.234		
No	163	137 (84.1)	1			
**Barriers to HIV testing at a clinic:**						
I do not know where to go						
No	130	103 (79.2)	1		1	
Yes	56	51 (91.1)	2.7 (1–7.4)	**0.057**	1.8 (0.6–5.2)	0.295
I do not have time to go to the clinic						
No	125	100 (80)	1			
Yes	61	54 (88.5)	1.9 (0. 8–4.7)	0.153		
It’s too far						
No	119	94 (79)	1		1	
Yes	67	60 (89.6)	2.3 (0.9–5.6)	**0.072**	2.2 (0.9–5.4)	0.097
Embarrassed discussing my sex life						
No	109	90 (82.6)	1			
Yes	77	64 (83.1)	1 (0.5–2.3)	0.922		
Embarrassed to ask for an HIV test						
No	72	56 (77.8)	1			
Yes	114	98 (86)	1.8 (0.8–3.8)	0.153		
Anxious when waiting for results						
No	128	100 (78.1)	1		1	
Yes	58	54 (93.1)	3.8 (1.3–11.3)	**0.018**	3.8 (1.3–11.3)	**0.018**
Embarrassed to be seen at clinic						
No	130	106 (81.5)	1			
Yes	56	48 (85.7)	1.4 (0.6–3.2)	0.490		
Concern over the confidentiality						
No	131	105 (80.2)	1			
Yes	55	49 (89.1)	2 (0. 8–5.2)	0.146		
Too afraid of having blood taken						
No	108	86 (79.6)	1			
Yes	78	68 (87.2)	1.7 (0.8–3.9)	0.182		
Fear of being diagnosed with HIV						
No	112	88 (78.6)	1		1	
Yes	74	66 (89.2)	2.3 (1–5.3)	**0.065**	1.4 (0.5–3.8)	0.473
I am feeling healthy						
No	107	91 (85.1)	1			
Yes	79	63 (79.8)	0.7 (0.3–1.5)	0.345		
Location of the brothel						
cluster 1	42	28 (66.7)	1			
cluster 2	21	21 (100)	1	–		
cluster 3	15	13 (80)	2 (0.5–8.3)	0.338		
cluster 4	27	27 (100)	1	–		
cluster 5	17	17 (100)	1	–		
cluster 6	49	40 (81.6)	2.2 (0.8–5.8)	0.105		
cluster 7	15	9 (60)	0.8 (0.2–2.5)	0.643		

* non-FSW

^#^ Brothel-based

### HIV self-test outcomes

Of the 188 men who accepted a HIV self-test and showed their result to the lay workers, there were 4 (2.1%) reactive results (all preferred fully assisted), of whom 2 gave a history of previous HIV testing. For these 4 cases, there was no evidence of subsequent attendance at the recommended referral HIV testing clinics at 2 months post HIV self-test.

### Post-test survey

All the men who accepted a HIV self-test completed the post-test acceptability survey (188 respondents). In the post-test acceptability survey, the most common responses in favour of HIV self-test were that men could test themselves and do so when they wanted, i.e. 89.8%. Most men, i.e. 75.1%, said there was nothing they did not like about the test. More than two-thirds of men, i.e. 77.6%, said that they trusted the result a lot or completely, with 22.5% reporting little trust. The majority of men, i.e. 90.4%, said it was likely or very likely that they would go to a doctor or clinic for further testing if they had a reactive HIV self-test result, including all men with a reactive HIV self-test result. (Table 4).

**Table 4:** Post-test acceptability survey findings on likes, dislikes about, and trust in HIV self-test among 188 respondents who accepted the HIV self-test

Items	n (%)
**What they liked about HIV self-testing**	
I could test myself	168 (89.8)
Gives results in 20–40 min	161 (86.1)
Convenient	157 (84)
No need to go to the doctor or clinic	157 (84)
No embarrassment	140 (74.9)
Allows me to test when I want	135 (72.2)
Does not require blood to be taken	126 (67.4)
Confidential	104 (55.6)
Saves time	102 (54.6)
Opportunity to test partner(s) at the same time	81 (43.3)
Others	1 (0.5)
**What they did not like about HIV self-testing**	
There was nothing I didn’t like	139 (75.1)
Concern over accuracy of the test	19 (10.3)
Not possible to have a full sexual health check at the same time	19 (10.3)
I don’t like being tested at brothels	14 (7.6)
Being supervised	4 (2.2)
Waiting 20–40 min for results	2 (1.1)
Difficult to perform	1 (0.5)
Other	1 (0.5)
**Whether they trusted in the results**	
Not at all	0 (0)
A little	42 (22.5)
A lot	140 (74.9)
Completely	5 (2.7)
**Likelihood to go to clinic for further testing after a reactive HIV self-test result**	
Unlikely/Very unlikely	18 (9.6)
Likely/Very likely	170 (90.4)

Also, among those completing the post-test acceptability survey (188 respondents), the majority of men indicated it would be likely or very likely they would test themselves (88.8%), or other men after the study finished (91%), and 63.3% would recommend a HIV self-test to partners. The places where men most often said they would like to purchase kits were chemists (88.8%), facilities (79.3%) or community organisations (70.7%). (Table 5).

**Table 5:** Post-test acceptability survey findings on likelihood of using a HIV self-test in the future among 188 respondents who accepted the HIV self-test

Likelihood to use HIV self-test in the future	Unlikely/ Very Unlikely n (%)	Likely/ Very likely n (%)
Likelihood to use HIV self-tests in the future to:		
Test yourself	21 (11.2)	167 (88.8)
Test a partner	69 (36.7)	119 (63.3)
Likelihood to recommend HIV self-tests to other men	17 (9)	171 (91)
Likelihood to purchase HIV self-tests from:		
Online/Internet	130 (69.2)	58 (30.9)
A chemist or pharmacy	21 (11.2)	167 (88.8)
A vending machine	138 (73.4)	50 (26.6)
A sexual health centre	39 (20.7)	149 (79.3)
A community organisation (e.g. YKP)	55 (29.3)	133 (70.7)

Nearly all, i.e. 97.3% men reported that it was easy for them to test themselves for HIV at brothels, and over 99% reported each individual step was easy or very easy. Most respondents (93%) said they would buy the kit at the price of IDR 50,000 ($US 3.5) or below (data is not presented on the table).

## Discussion

This study is the first to investigate the acceptance of HIV self-testing when offered to MWPS in brothels by lay health providers. Men attending brothels in Bali, Indonesia had a high level of acceptance of HIV self-test, with 64.6% of men offered an HIV self-test agreeing to have it. Nearly all preferred to be assisted when they conducted the self-test and of these, most preferred to be fully assisted consistent with the WHO definition, while 16.1% opted to be partially assisted which allowed them to read their results privately. Acceptability of the procedure was high, with most men reporting they liked the fact that they could test themselves, they found performing each step of the HIV self-test procedure easy and they were interested in future use. The level of HIV testing uptake achieved by offering a HIV self-test was around 6–7 times higher than current levels and findings from a past survey in 2015 in the same population in Bali [45] and 37 times higher than HIV testing rates among other high-risk men in Indonesia, including truck drivers, moto-taxi drivers, dockworkers and seafarers [44].

Understanding barriers (structural or attitudinal) to HIV testing at clinics, can inform the design of HIV self-testing programs. Our study demonstrated that offering a HIV self-test by a lay provider at the brothel seemed to overcome some of barriers raised by the men in the survey with regards to clinic-based HIV testing such as feeling embarrassed to ask for an HIV test at the clinic, embarrassment attending the clinic, afraid of having blood taken, and distance to clinic. Men were more likely to accept HIV self-test if they stated feeling embarrassed to ask for an HIV test at the clinic as one of the barrier to testing, which is inline with previous study conducted in this setting [48], and indicating the potential roles of HIV testing, as has been endorsed by WHO, to improve HIV testing access among those who are reluctant to visit the VCT clinics and ask for a HIV [25].

Men were less likely to accept HIV self-test if they feared being diagnosed with HIV, i.e. 55.2% of men who reported a fear of being diagnosed with HIV were wiling to have a self-test, compared with 72.7% of men who did not report a fear. Fear of being diagnosed with HIV was also found to influence participant decisions in declining HIV self-test in a study in Cape Town [65]. It is likely that the fear of a HIV diagnosis was related to HIV stigma, which may be reduced by the ability of HIV self-test to normalise the testing procedure [66]. Efforts which help make the HIV self-testing more common and to alleviate HIV related stigma are thus paramount in this setting.

Most men preferred assisted HIV self-testing, even though the lay workers had minimal training, suggesting that lay providers could play an important role in HIV self-test models among MWPS in the future, as recommended in WHO’s consolidated guidelines on HIV testing [25]. Although there are at least 6924 VCT clinics in Indonesia in 2019 [47], there are over 5 million MWPS, highlighting the importance of utilising other strategies to reach those who might be unable or unwilling to seek testing at the clinic [25]. Involving lay workers in HIV prevention has reportedly led to substantial increases in the use of condoms use at brothel settings compared to a few decades ago [45]. The same success is possible for HIV self-test programs if support is provided for these lay workers.

The majority of men preferred fully assisted HIV self-testing with supervision during the reading of the test result. Men were more likely to prefer fully assisted HIV self-test if they reported anxiety when waiting for results as a barrier to HIV testing at the clinic, so the presence of someone assisting with the process may have overcome these concerns [67], particularly considering the majority of men had never HIV tested previously. The high acceptance of assisted HIV self-testing among first-time testers has also been demonstrated in other studies such as those adolescents in Mozambique [68]. Two third of the participants in that study had never HIV tested previously, and over 75% of the participants chose directly assisted HIV self-testing [68]. The proportion requesting fully assisted testing might decrease as HIV self-testing becomes more common.

The analysis found very few associations between the questions asked and uptake of HIV self-test. The variables came from a brief health survey, and thus men were not asked about psychosocial or contextual factors related to their visit to the brothel that may have influenced their decision to have a self-testing. We deliberately kept the health survey brief and unrelated to self-testing to prevent the men from losing their interest in participating in the study. What the survey was able to show that ethnicity, age and sexual risk practices did not influence their decision to have a self-test, which is important information when scaling up the technology. We believe their willingness to have a self-test was related to convenience and privacy as demonstrated in our previous in-depth interviews [48].

The study highlighted a number of issues to consider for future HIV self-testing programs in MWPS in Indonesia. The first being the access points: nearly all men liked accessing HIV self-tests at brothels, but when asked about other potential ways, they cited a chemist as their preferred place to purchase a HIV self-test, consistent with long-standing practices in Indonesia and other Asia settings of self-purchasing medication for prevention and treatment of sexually transmitted infections [69, 70], and other treatments from pharmacies [71]. This might be due to the fact that sexually transmitted infections (STIs), including HIV, are highly stigmatised, and accessing health care through private pharmacies or street vendors provides easy access to a service with little fear of being interrogated with embarrassing questions [72]. Second, about a third of men reported ‘little trust’ in the results, suggesting the need for more community education about HIV self-tests and their accuracy. Third, although the majority of men (90.4%) reported it was likely they would go to a doctor or clinic for further testing if they had a reactive HIV self-test result, in reality none of the four men with a reactive result attended for further testing at the recommended referral HIV testing clinic over a two-month follow-up period. Two of the four men had been tested in the past, but were not asked about the results of their past HIV test, so it is possible they already knew they had HIV infection, or were already in care [29], and did not feel the need to have another test at the clinic. It may also be that the four men did link outside the study clinic.

Linkage to care is a complex matter, and its measurement is challenging [73]. Various factors might also influence poor linkage, including stigma and the population being tested [73]. When scaling up HIV self-testing programmatically, strategies to improve linkage to care should be considered and evaluated such as phone calls, home visits from health care workers, including taking the confirmatory test to the home, and financial incentives [74–76].

With a program supported by the Bill & Melinda Gates Foundation in 50 low- and middle-income countries, the cost of the OraQuick® HIV self-test is now as low as US$ 2 per test kit [77]. Given that most of the respondents in the current study also preferred the price of lower than $3.5, the costs of self-tests needs to be considered if introducing HIV self-testing in this setting.

There are a few other limitations to consider also when interpreting the study findings, including that the data were collected via an interview rather than self-completion due to low education levels, and may be affected by social desirability bias [78, 79]. However, given the similarities of the findings with other studies, for example, the findings on previous testing rates and barriers to HIV testing, it may indicate otherwise. Also, participants of this study were recruited from brothel areas and might not be representative of all MWPS, particularly those who access non-brothel-based FSWs. Finally, the 2.1% HIV prevalence found in the current study may be an underestimation, as it is possible that some men visiting the brothels who were already HIV positive decided not to participate in the study; or an overestimation as we cannot rule out false positives. However, an overestimation is unlikely considering the high specificity of the test [80].

## Conclusions

In conclusion, our study demonstrated that the majority of MWPS who were offered a HIV self-test by lay providers onsite at brothels would accept a HIV self-test, with most men preferring assisted to unassisted testing. HIV self-self test is not currently available free in the country. The high uptake of HIV self-test indicates its considerable potential to improve the rate of HIV testing among MWPS in Indonesia, either through lay workers’ distribution at brothels or more broadly through purchasing through chemists as indicated in the survey, something that should be considered for HIV testing policy and programming in Indonesia. Future implementation research should assess the role of both lay workers at brothels and chemists in reaching MWPS in Indonesia, and consider novel strategies to improve and measure linkage to care. Future research should assess costs and the scalability of this model of HIV testing.

## Data Availability

The dataset generated and/or analysed in the current study is not publicly available due to it contains potentially sensitive information. Requests for the data may be sent to Luh Putu Lila Wulandari (putuwulandari@gmail.com, or lwulandari@kirby.unsw.edu.au).

## Abbreviations

FSW: Female sex worker
MWPS: Men who purchase sex
STIs: Sexually transmitted infections
VCT: Voluntary counselling and testing
YKP: Yayasan Kerti Praja
NGO: Non-government organization
